# Tandem fluorescent Halo-GFP reporter for quantitative and time-resolved monitoring of organelle and protein delivery to lysosomes

**DOI:** 10.1080/27694127.2022.2061679

**Published:** 2022-04-29

**Authors:** M. Rudinskiy, M. Molinari

**Affiliations:** a Università della Svizzera italiana (USI), Faculty of Biomedical Sciences, Institute for Research in Biomedicine, CH-6500 Bellinzona, Switzerland; b Department of Biology, Swiss Federal Institute of Technology, CH-8093 Zurich, Switzerland; c School of Life Sciences, École Polytechnique Fédérale de Lausanne, CH-1015 Lausanne, Switzerland

**Keywords:** Alpha-1 antitrypsin Z variant (ATZ), autophagy, endolysosomes, endoplasmic reticulum autophagy (ER-phagy/reticulophagy), ER-to-lysosome-associated degradation (ERLAD), recov-ER-phagy, quantitative and time-resolved assays, tandem fluorescent reporter

Autophagic events contribute to cellular homeostasis by ensuring constitutive and regulated degradation of excess/faulty/damaged/aged macromolecules and portions of organelles, and by recycling the products of degradation into basic building blocks that fuel essential cellular processes. They rely on delivery of the material to be cleared from cells to lysosomes (or vacuoles in yeast and plants). Defects in these catabolic pathways, in lysosomal biogenesis and activity are associated with cell and tissue dysfunction and cause human diseases. Accurate and quantitative monitoring of protein and organelle delivery to degradative compartments is crucial to mechanistically dissect intracellular pathways including those regulating autophagy, intracellular trafficking, lysosome biogenesis, and protein and organelle turnover. It is instrumental to develop and evaluate for therapeutic purposes pharmacological modulators of the pathways. In our latest paper, we describe a novel tandem fluorescent Halo-GFP reporter for quantitative and, notably, for time-resolved analyses of organelle and protein delivery to lysosomes.

## Monitoring autophagy

Autophagic events have historically been monitored by assessing: i) the extent and stability of Atg8/LC3 lipidation resulting in a shift in the protein’s electrophoretic mobility, ii) the formation of cytoplasmic Atg8/LC3 puncta by fluorescence microscopy and their colocalization with macromolecules or organelles to be removed from cells, iii) the turnover of autophagic substrates, and iv) their accumulation within lysosomes/vacuoles in cells exposed to inhibitors of acidic hydrolases or of compounds that neutralize the lysosomal/vacuolar pH.

## pH-sensitive proteolysis, pH-sensitive fluorescence shift and tandem reporters

The strong acidity of the lysosomal environment has been exploited to signal lysosome-related events in a pH-specific manner. pH-sensitive reporters have been appended to substrate proteins or to organelle marker proteins to signal their arrival within acidic degradative compartments. For example, when delivered within highly acidic lysosomes, the fluorescent protein Keima shifts to a longer-wavelength excitation and the mCherry Cleavage from the ER (CCER) reporter releases acid and protease-resistant mCherry polypeptide fragments detectable in western blot.

More inspiring for our study, tandem-fluorescent reporters contain two fluorescent components, the low pH-resistant mCherry polypeptide (or the low pH-resistant red fluorescent protein, RFP) and the low pH-sensitive green fluorescent protein (GFP). These reporters combine red and green fluorescence emission, when located outside lysosomes. Upon arrival in the acidic degradative compartments, mCherry and RFP maintain their fluorescence, whereas the green fluorescence is quenched. This results in red-only emission within lysosomes that can be visualized with light microscopy or flow cytometry.

In our latest publication [1], we present a tandem fluorescent Halo-GFP reporter, where the low pH-resistant mCherry/RFP part of conventional tandem fluorescent reporters has been replaced with a low pH-resistant HaloTag. HaloTag is an engineered, enzymatically inactive bacterial dehalogenase that covalently and irreversibly binds cell-permeable ligands modified with a large variety of functional groups including fluorescent, surface and reactive molecules.

## Halo-GFP reporter for quantitative analyses of delivery of ER portions to active lysosomes by imaging, biochemical and flow cytometry approaches

**Figure 1.**
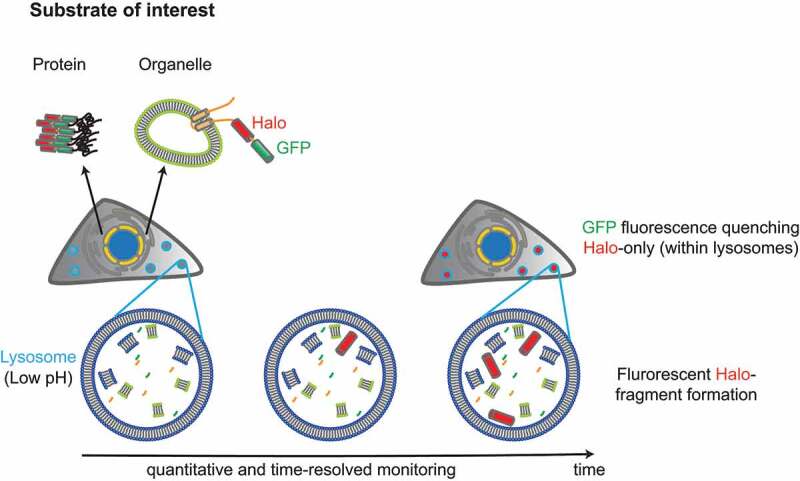
Delivery of Halo-GFP-tagged substrates to the low-pH environment of lysosomes manifests in the quenching of GFP fluorescence and subsequent cleavage and accumulation of fluorescent Halo fragments inside the degradative lysosomal compartments. Quantitative and time-resolved monitoring of lysosomal delivery events is achieved by incorporating a series of fluorescent and non-fluorescent HaloTag ligands to label the population of newly synthetized reporter proteins and follow their delivery to lysosomes.

We first validated the Halo-GFP reporter in two independent models of selective lysosomal turnover of the ER (reticulophagy/ER-phagy): i) delivery of excess ER portion to endolysosomes during cellular recovery from ER stress (aka recov-ER-phagy) mimicked by the overexpression of the reticulophagy receptor SEC62-Halo-GFP, and ii) turnover of ER portions containing misfolded proteins (aka ER-to-lysosome-associated degradation, ERLAD) by using a misfolded polymerogenic Z variant of SERPINA1/alpha-1 antitrypsin (Halo-GFP-ATZ) as a model ERLAD client. Consistent with the loss of GFP fluorescence at low pH, lysosomal delivery of ER portions decorated with SEC62-Halo-GFP or containing misfolded Halo-GFP-ATZ result in Halo-only fluorescent puncta in LAMP1-positive endolysosomal compartments (Figure 1). Furthermore, the relative stability of the HaloTag portion of the chimeric polypeptides in the highly hydrolytic lysosomal lumen generates fluorescent Halo fragments that are separated in SDS-PAGE. As an important difference with fluorescent mCherry and RFP, the fluorescence of the Halo fragment is generated by a small molecule covalently associated with the Halo polypeptide, which retains fluorescence during sample processing for gel electrophoresis; hence, it can be directly quantified in gels. Finally, lysosomal delivery of the Halo-GFP reporter proteins results in a fluorescent shift caused by the persistence of the Halo ligand fluorescence on quenching of the GFP fluorescence at low pH, which is detectable in flow cytometry on a whole-cell scale.

## Adding time-resolution to fluorescence-based investigation of lysosomal pathways

In contrast to mCherry and RFP, the HaloTag polypeptide is not fluorescent *per se*. Thus, Halo-GFP reporters display only GFP fluorescence, until the cell culture media is supplemented with the fluorescent HaloTag ligands (available in various flavors). Thus, the pH-resistant HaloTag fluorescence can be activated on demand, and fluorescence incorporation in the newly synthesized HaloTag reporter can readily be interrupted by replacing the fluorescent ligand, with a non-fluorescent, ”black” ligand, in the culture media. This, combined with the loss of GFP fluorescence upon the reporter’s arrival in acidic degradative compartments, allows fluorescent labeling of a discrete population of newly synthetized reporter proteins, whose fate is visualized by microscopy or biochemically during increasing chase times.

All in all, fluorescent pulse-chase of newly synthetized SEC62-Halo-GFP and Halo-GFP-ATZ reveal the kinetics of recov-ER-phagy and ATZ ERLAD that were previously only observed in cells treated with either lysosomal inhibitors (that nonspecifically perturb multiple cellular pathways) or by performing pulse-chases with radioactively labeled amino acids, which requires special equipment and training.

## Future perspectives

Halo-GFP is a versatile tandem fluorescent tag to be appended at the N or at the C terminus of reporter proteins-of-interest. In our *proof-of-principle* analyses, it has been used to assess lysosomal delivery of ER portions or of misfolded proteins trapped in ER-derived vesicles in a time-resolved manner. We envision the use of Halo-GFP as a tag to label protein markers of other organelles such as mitochondria, peroxisomes, or nuclei, to label autophagy gene products such as LC3 and monitor autophagic flux, or to modify components of multimeric complexes such as ribosomes, nuclear pore complexes, translocation channels and others to assess their biogenesis, assembly, disassembly, regulated activation or inactivation and turnover. Changes of biophysical/biochemical features of the Halo-GFP tandem fluorescent tag delivered within acidic and hydrolytic compartments can be quantified with a plethora of antibody-free, biochemical, flow cytometry, imaging techniques available in cell biology labs, including time-resolved transmission electron microscopy. This latter use relies on the availability of cell permeable HaloTag ligands that are enzymatically active and can generate electron-dense reaction products of 3,3’-diaminobenzidine/DAB. We also envision applications of the Halo-GFP reporter in translational research for the characterization and evaluation of pharmacological therapies against diseases caused by defective protein folding and/or defective lysosomal trafficking or lysosomal function. In this context, flow cytometry and imaging pipeline automation and machine-learning approaches like LysoQuant, a deep learning approach for unbiased and automated image analyses developed in our lab, will greatly widen the capabilities for quantitative and time-resolved mechanistic and translational studies of lysosome-driven cellular pathways.
